# New Target(s) for RNF43 Regulation: Implications for Therapeutic Strategies

**DOI:** 10.3390/ijms25158083

**Published:** 2024-07-24

**Authors:** Jeetendra Kumar Nag, Priyanga Appasamy, Hodaya Malka, Shoshana Sedley, Rachel Bar-Shavit

**Affiliations:** Sharett Institute of Oncology, Hadassah Medical Center, Hebrew University, Jerusalem 91120, Israel; nagj3@ccf.org (J.K.N.); priyanga.appasamy@mail.huji.ac.il (P.A.); hodaya.malka@mail.huji.ac.il (H.M.); shanisedley@gmail.com (S.S.)

**Keywords:** β-catenin stabilization, colon cancer, G-protein-coupled receptors (GPCRs), protease-activated receptors (PARs), proteases, RNF43/ZNRF3

## Abstract

Cancer cells depend on specific oncogenic pathways or present a genetic alteration that leads to a particular disturbance. Still, personalized and targeted biological therapy remains challenging, with current efforts generally yielding disappointing results. Carefully assessing onco-target molecular pathways can, however, potently assist with such efforts for the selection of patient populations that would best respond to a given drug treatment. RNF43, an E3 ubiquitin ligase that negatively regulates Wnt/frizzled (FZD) receptors by their ubiquitination, internalization, and degradation, controls a key pathway in cancer. Recently, additional target proteins of RNF43 were described, including p85 of the PI3K/AKT/mTOR signaling pathway and protease-activated receptor 2 (PAR_2_), a G-protein-coupled receptor that potently induces β-catenin stabilization, independent of Wnts. RNF43 mutations with impaired E3 ligase activity were found in several types of cancers (e.g., gastrointestinal system tumors and endometrial and ovarian cancer), pointing to a high dependency on FZD receptors and possibly PAR_2_ and the PI3K/AKT/mTOR signaling pathway. The development of drugs toward these targets is essential for improved treatment of cancer patients.

## 1. Introduction

The dogma of targeted cancer therapy relies on the development of drugs directed to mutated target genes that lead to dysregulated gene function and cancer. While cancer cells depend on specific oncogenic pathways or result from a given genetic alteration, targeted therapy remains a challenge. Targeted therapies led to a cascade of cancer drug development; for example, between 2006 and 2015, over 3000 phase 1 clinical trials in oncology started. Despite innovative know-how in genomics technology, only 5.1% of the drugs that initiated phase 1 continued to Food and Drug Administration (FDA) approval [1]. The high level of failure of such efforts is related to the interplay between the dose needed for therapy and toxicity, namely, the therapeutic index. In addition, resistance to a given drug that develops with time has been shown to affect outcomes. These serious limitations underscore the ongoing need for alternative targeting routes and key target genes.

Wnts/β-catenin signaling plays a key role in the morphogenesis of embryonic development, adult homeostasis, and cancer [2,3]. The Wnt family originated from an ancient protein that appeared during metazoan evolution. No Wnts have been found in unicellular eukaryotes or in other primitive organisms thought to represent common ancestor animals [4]. Hence, the ability of cells to communicate via extracellular signals, as implicated in cell–cell interactions, is a key element of multicellular organisms. Proteins such as Wnts are responsible for such communication signals. Wnts are secreted glycoproteins enriched in cysteine residues (e.g., 22 cysteines in some Wnts), which may be palmitoylated, as occurs with Wnt3A cysteine 77 [5]. To enable lipid addition to Wnts, an acyltransferase encoded by the *Drosophila porcupine (porc)* gene uniquely acts to add an acetyl group to Wnts. The molecular mechanism depicting a negative control layer in Wnt signaling is developing, clarifying the tight regulation required for the precise coordination of the Wnt cancer pathway. In the search for new therapeutic strategies, it is essential to thoroughly comprehend which cancers are driven by the Wnt-induced β-catenin stabilization pathway or by other inducers of β-catenin stabilization, such as the PAR-guided β-catenin pathway.

**Figure 1:**
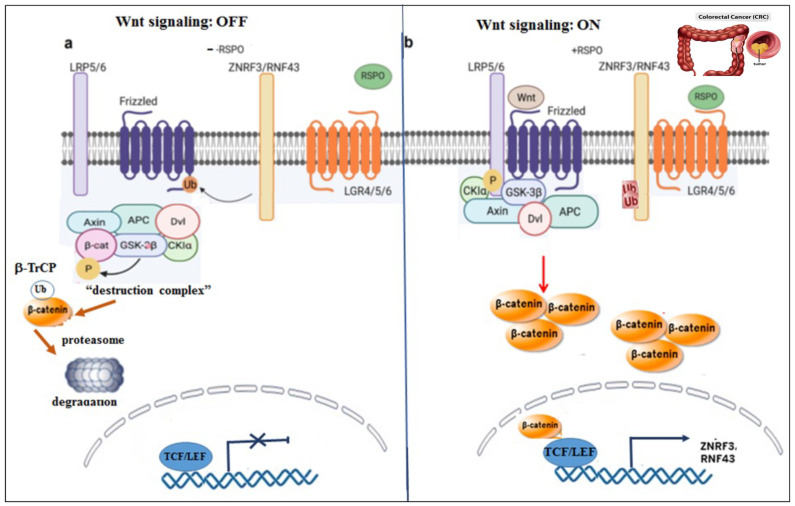
Wnt signaling On–Off. A schematic model portraying Wnt signaling On–Off states. (**a**) OFF status. When Wnt ligands are absent, the “destruction complex” actively acts to tag β-catenin (e.g., phosphorylation) for degradation via the proteasomal compartment. FZDs are ubiquitinated, internalized, and degraded in the endosome. (**b**) ON status. In the presence of Wnt ligands, the “destruction complex is dismantled and recruited to the cell membrane, and β-catenin is accumulated. In the presence of RSPO, no ubiquitination of FZDs takes place.

Upon binding of Wnt to a cell surface receptor, a signal is delivered into the cytoplasm, reflected as the level of β-catenin stabilization. This is followed by β-catenin transfer into the cell nucleus, where it acts as part of a transcription factor complex that, in turn, affects a set of genes. Primary components involved in this well-studied pathway include frizzled (FZD) receptors and their co-receptor LRP5/6. Upon Wnt binding, FZD receptors and LRP5/6 form a complex that recruits disheveled (DVL) to initiate cell signaling. This leads to the expression of a gene signature driven by β-catenin and Lef/Tcf-mediated transcription [6,7,8]. In the absence of Wnt binding, β-catenin is tagged by the “destruction complex” for proteasomal degradation [9]. The “destruction complex” is composed of axin, APC, and the two kinases, GSK3β and CKIα. Upon tagging by this complex, β-catenin is primarily phosphorylated on serine 45 (S45) by CK1α, enabling the subsequent phosphorylation of S33, S37, and T41 (Threonine 41) by GSK3β. Upon phosphorylation, β-catenin is ubiquitinated by the ubiquitin ligase β-TrCP for proteasomal degradation. In contrast, when Wnts are bound to FZD-LRP5/6 co-receptors, the “destruction complex” is recruited to the cell membrane via axin’s association with DVL. In this latter scenario, the system is incapable of tagging β-catenin, and it is no longer ubiquitinated or degraded (Figure 1).

Ring finger protein 43 (RNF43) and ring finger protein 3 (ZNRF3) or RNF43/ZNRF3, act as a negative regulator of the Wnt signaling pathway and are associated with FZD receptors, leading to receptor ubiquitination, internalization, and lysosomal degradation. For this, RNF43/ZNRF3 surprisingly co-associates with FZDs via recruitment of the Wnt-signaling component DVL [10]. RNF43/ZNRF3 are closely related proteins that belong to the RING family of E3 ubiquitin ligases, which transfer ubiquitin from the E2 enzyme to a substrate. The specificity of RNF43/ZNRF3 among the family is their substrate FZDs, the Wnt signaling receptors, tagged by ubiquitin for degradation. Both proteins comprise an amino-terminal extracellular region of 39% sequence conseservation between the two proteins, a transmembrane region, and a cytoplasmic tail, including the RING-type E3 ubiquitin ligase.

Several labs (e.g., Hao et al., 2012; Koo et al., 2012; Tsukiyama et al., 2015; [11,12,13]) have independently identified ZNRF3 and its functional homolog RNF43. Their findings were based on the fact that the level of FZDs is mainly regulated and stabilized by deubiquitinating enzymes (DUBs); ubiquitin-specific protease 8; USP8 [14]; and USP6 [15], which remove ubiquitin from FZDs. FZD/Wnt signaling regulation by RNF43/ZNRF3 occurs by a striking and complex mechanism that works together with the intestinal R-spondin [11,12,16,17,18]. They both are type I single transmembrane proteins of a distinctive structural organization. They comprise two functional domains, a protease-associated (PA) domain in the extracellular region for the interaction with other proteins and an intracellular ring finger (RING) domain for tagging proteins with ubiquitin. They act on both canonical and non-canonical Wnt signaling pathways [11,12,13].

While DVL is known to be a Wnt-induced signal protein that positively promotes Wnt/β-catenin signaling, it also induces the degradation of FZDs when recruited to FZDs-ZNRF3/RNF43 as an adaptor protein. It appears that physical association between the FZD and the DEP domain of DVL is required for the ZNRF/RNF43-mediated degradation of FZDs. Indeed, *DVL* knockout cells showed greatly increased cell surface FZD levels. As such, DVL plays a dual role, acting to recruit ZNRF3/RNF43 to FZDs for their degradation so as to maintain proper control of the Wnt/FZD pathway and serving as a signal protein that induces the β-catenin stabilization pathway. Importantly, ZNRF3 and RNF43 are located in stem cells, including ISCs, where they act to diminish and regulate uncontrolled stem cell proliferation.

The rescue of FZD degradation by RNF43/ZNRF3 takes place in the presence of R-spondins, namely, ligands of the leucine-rich repeat-containing G-protein-coupled receptor (LGR5), a member of the LGR4 and LGR6 families. Significantly, LGR5 is the bona fide intestinal stem cell (ISC) marker [19]. Certain genetic mutations in *RNF43* lead to a loss of RNF43 function (LOF), whereby the mutant proteins lose their ability to ubiquitinate and, consequently, degrade FZDs. Instead, these mutants now facilitate the β-catenin stabilization pathway. Mutations in *RNF43* are frequently found in colon, pancreas, stomach, ovary, endometrial, and liver cancers [20,21,22,23,24,25,26]. This suggests that RNF43 corresponds to an important “dual sword” junction factor that assumes a significant role in preserving tissue homeostasis so as to negate a pathological status; at the same time, *RNF43* mutations induce tumorigenesis. Cancers that harbor Wnt mutants are divided as follows. Mutations in genes encoding Wnt pathway components within the cytoplasmic compartment, such as those encoding APC and CTNNB1 (e.g., β-catenin), are categorized as leading to constitutive Wnt pathway activation and are ligand (Wnt) independent (i.e., they do not require Wnt ligands). In contrast, tumors presenting R-spondin (RSPO) or *RNF43* mutants require exogenous Wnt ligands to induce Wnt signaling (i.e., ligand dependent).

This review focuses on events leading to the appearance of the negative regulatory layer of the β-catenin stabilization pathway in cancer that is critical for the precise coordination and proper homeostasis of β-catenin stabilization, yet which is also implicated in development and tissue homeostasis. The purpose of the present review is to describe additional targets of RNF43 and the RNF43p.G659fs mutant, such as PAR_2_ and the PI3K/AKT/mTOR signaling pathway, respectively. The development of drugs toward these targets is important and needed for the effective treatment of cancer patients.

## 2. Post-Translational Regulation of RNF43/ZNRF3

Ubiquitination refers to the conjugation of ubiquitin to a selected protein. This is a multistep cascade event initiated by E1, which is a ubiquitin-activating enzyme. This is followed by the actions of E2, a ubiquitin-conjugating enzyme, and culminates with E3 ligase, responsible for the final delivery of ubiquitin to a specific protein substrate. In general, ubiquitin binds to a lysine residue of the protein substrate. In eukaryotes, this major protein degradation pathway is called the ubiquitin–proteasome system (UPS).

It is well established that FZDs undergo lysosomal trafficking via ubiquitination in order to ensure the delicate and dynamic homeostasis of cell surface FZD levels. Ubiquitination induces the internalization of FZDs and co-receptor LRP5/6 toward lysosomal degradation via the endosome. Post-translational modifications transpire following the attachment of matching pairs of E3 ligases (i.e., ubiquitinating enzymes) and DUBs [27,28]. DUBs, such as USP8, induce the recycling of FZDs to the cell surface membrane, thus enhancing the β-catenin stabilization pathway [14]. To control RNF43/ZNRF3 levels, negative regulation is mediated via auto-ubiquitination and membrane clearance, which is mediated by R-spondin binding to LGR4-6. Once RNF43/ZNRF3 is internalized and no longer appears on the cell surface, it leaves FZDs available, resulting in enhanced Wnt signaling.

Gieble et al. unmasked another piece in the puzzle of RNF43/ZNRF3 regulation [29] when they elegantly demonstrated that USP42 deubiquitinates RNF43/ZNRF3, thus negating the actions of the R-spondin–LGR5 axis. It was shown that USP42 associates with the DVL-interacting region (DIR) of ZNRF3, which reinforces the LGR-ZNRF3-RSPO complex, leaving cell surface RNF43/ZNRF3 intact and functionally active. Therefore, in the presence of USP42, ongoing induced degradation and turnover of FZDs continue, impacting Wnts signaling [29]. USP42 maintains the LGR5/RNF43/ZNRF3 complex at the plasma membrane through its deubiquitinating ability by eliminating RSPO- and ubiquitin-mediated internalization of RNF43/ZNRF3. This is strengthened in cells expressing ZNRF3ΔRing. Lacking the RING domain, these mutants are incapable of ubiquitination and hence display a high degree of ZNRF3ΔRing membrane localization and enhanced association with R-spondin and LGR5 relative to wild-type (*wt*) ZNRF3. The increased relevance of USP42 to the Wnt/FZD pathway also stems from the fact that USP42 associates via the DVL interacting region of ZNRF3. Together, these observations point to the central function of USP42 in regulating the Wnt/FZD pathway.

On the other hand, a truncation mutation in RNF43 found in cancer patients causes increased β-catenin stabilization and enhanced Wnt signaling. Interestingly, it does not occur via the LOF RNF43 mutation. In fact, the mutation of RNF43 at residue R519X gives rise to a shortened form of RNF43. This truncated RNF43 associates with CK1 kinase, a main component of the Wnt “destruction complex”, thereby impacting β-catenin stabilization. Once CK1 binds to the truncated RNF43, it is linked to the cell membrane and is no longer capable of mediating the priming phosphorylation step of β-catenin tagging for proteasomal degradation. As a result of CK1 sequestration via R519X, Wnt signaling is promoted, leading to increased β-catenin stabilization and transcriptional activity [30]. TCGA PanCancer atlas studies by Xu Y et al. [31] demonstrated high levels of RNF43 genetic alterations in endometrial, stomach, and colon cancers.

Another component found to be involved in the negative regulation of RNF43/ZNRF3 is the protein tyrosine phosphatase receptor-type kappa (PTPRK). The PTPRK is a Wnt inhibitor in both human cancer cells and is found in the Spemann organizer of *Xenopus* embryos [32]. The actions of the PTPRK are mediated by dephosphorylation of the tyrosine-containing motif, which is known to play a role in the endocytosis of transmembrane receptors. Sequencing and alignment of the intracellular portion of ZNRF3 uncovered an identical cluster of four tyrosine residues, called the “4Y” motif (Y465, Y469, Y472, and Y473), which is highly conserved across vertebrates. A construct in which the “4Y” domain is deleted showed an increased level of ZNRF3 in the membrane, relative to that seen with wild-type ZNRF3. This points to the significant role played by tyrosine phosphorylation within ZNRF3 in regulating endocytosis. Accordingly, a model stating that the 4Y domain of ZNRF3 is an endocytic signal that induces ZNRF3-Wnt receptor co-internalization has been suggested. When an unknown tyrosine kinase phosphorylates these tyrosines, it stops ZNRF3-FZD internalization, resulting in increased Wnt signaling. In contrast, in the presence of the PTPRK, which dephosphorylates the 4Y domain, considerable ZNRF3-FZD endocytosis takes place, resulting in Wnt signaling inhibition.

Early publications on RNF43 appeared in 2004 by a Japanese group stating that RNF43 is an oncoprotein highly expressed in colorectal cancer (CRC) [33]. At the same time, Uchida N et al. [34] published that RNF43 is a promising candidate for Tumor-Associated Antigen (TAA). RNF43 was allocated following a genome-wide screening of cDNA microarray profiling that contained 23,040 genes. RNF43-derived peptides of TAA were tested for the induction of cytotoxic T-lymphocytes (CTLs) and CD8+ T cells, specific for tumor cells. This publication was followed by an additional article in 2010 reporting on phase I clinical trial testing the safety and immune responses of peptide vaccines derived from RNF43 and 34-kDa translocase of the outer mitochondrial membrane (TOMM34), which were upregulated in more than 80% of CRC. The vaccine was administered in combination with chemotherapy in patients with metastatic colorectal cancer. The trial showed that vaccination with the two colorectal cancer-specific peptides in combination with UFT/LV chemotherapy is well tolerated and can induce CTL response in least in 95% of the patients [35]. Since then, there has been no follow up to the direction of RNF43 as TAA-inducing CD8+ T-lymphocytes.

## 3. Phospho-Regulation of RNF43

**Figure 2:**
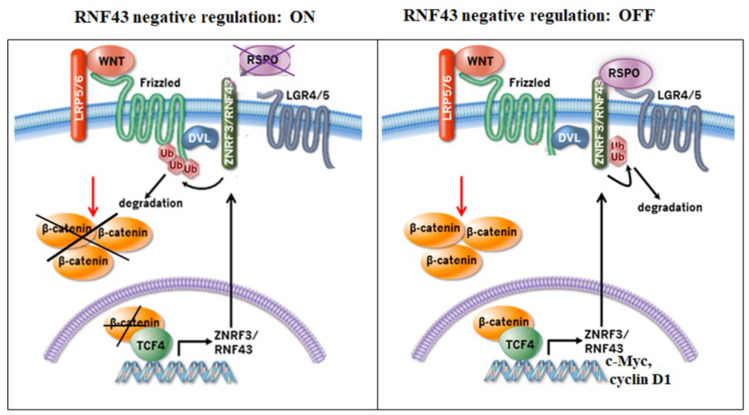
Wnt signaling negative regulation of RNF43/ZNRF3: ON-OFF. When RNF43/ZNRF3 ubiquitinate FZDs, they internalize and degrade via the endosome compartment. Hence, no β-catenin accumulation takes place. In the presence of RSPO ligands of LGR4/5 receptors, they bind to RNF43/ZNRF3 and LGR4/5. RNF43/ZNRF3 are ubiquitinated and internalized. FZDs no longer undergo ubiquitination and consequently the Wnt signaling is on.

Significant core proteins of Wnt signaling are often mutated in different types of tumors [36]. Indeed, truncation or missense mutations in *rnf43* that harm the negative regulation of Wnt signaling have been found in cancer [15,28]. While these tumors require Wnt for growth, they do not require Rspo [13]. Indeed, RNF43, encoded by a downstream target gene of the Wnt/β-catenin pathway [37], functions as a central negative feedback control to maintain proper Wnt signaling and prevent excess activity of the Wnt/β-catenin pathway (Figure 2). RNF43 is only expressed in the intestinal stem cells (ISCs) that are localized within the colon crypts [12,38]. Once RNF43 is synthesized, it is transported to the cell membrane via the intracellular trafficking as nascent non-modified RNF43.

**Figure 3:**
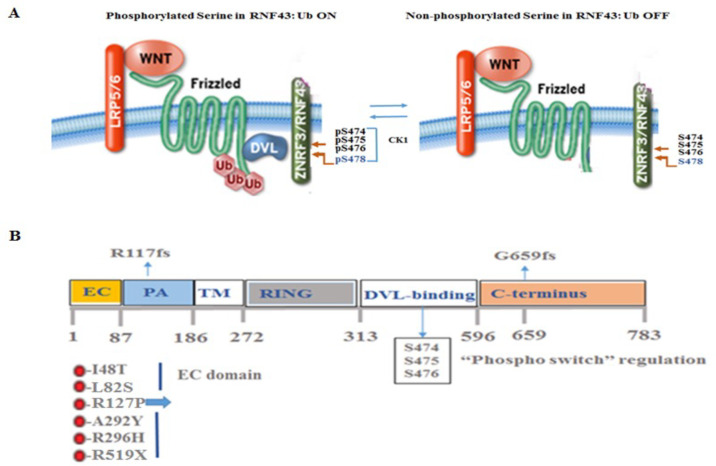
The “phosphorylation- switch” regulates RNF43 function and scheme of RNF43. (**A**) Model portraying key phosphorylation sites in RNF43. (**B**) The scheme of RNF43 showing specific regions, mutations, and serine phosphorylation sites.

Numerous cancer-linked *RNF43* mutations affecting regions outside those encoding essential RNF43 domains, such as the FZD-binding site (found in the PA domain of the N-terminal extracellular domain) and the RING ubiquitination site (found in the RING domain within the cytoplasmic part) possibly point to the existence of an extra mode of RNF43 control. Certainly, in addition to the DVL-binding domain located in the RNF43 cytoplasmic domain, additional amino acids, namely, residues 442-478 in the C-terminal tail, were found to be significant for controlling RNF43 [39]. Within this region, two serine-rich regions (SRRs) are found, namely, SRR-1, located between residues 442 and 449, and SRR-2, positioned between residues 466 and 478. The SRR-2 region is preserved in RNF43 and ZNRF3 but not SRR-1. In SRR-2, three consecutive serine residues (S474, S475, and S476) are found as a triplet and are vital for the RNF43-mediated degradation of FZDs. It has been demonstrated that these serines can be phosphorylated. When serine residues are replaced by amino acids that mimic the phosphorylated form of serine, such as aspartic acid (3SD) or glutamic acid (3SE), negative regulation of RNF43 is maintained. In contrast, mutations that introduced an alanine (3SA) or threonine (3ST) able to inhibit phosphorylation led RNF43 to lose its negative regulation of FZDs compared to wild-type RNF43. Overall, this suggests that phosphorylation of the serine triplet is required to mediate the negative control of RNF43. This was demonstrated via Phos-tag SDS-PAGE, whereby phosphorylated proteins were identified by a band shift. Similarly, a loss of phospho-RNF43 (p-RNF43) is seen in cells exhibiting mutated serine triplets, expressing alanines (3SA), relative to wild-type RNF43. Furthermore, it was shown that S478 is vital for the control of RNF43. It is sufficient to substitute S478 with alanine to inhibit RNF43 function, as shown by TOP*flash* transcriptional activity, while substitution with aspartic acid or glutamic acid (mimetics of serine phosphorylation) induced the negative function of RNF43 (Figure 3A,B). The “phospho-switch” of RNF43 signals ON, while the 3SA mutant signals switch OFF. While tumors are being continuously propagated when RNF43 is switched OFF in the presence of 3SA (which cannot be turned on), under conditions where the switch is ON with the serine mimetic 3SD (incapable of turning off), normal intestinal tissues are eventually no longer maintained due to extensive FZD clearance (Tskiyama T et al. 2021 [2]).

Another aspect of RNF43-mediated negative regulation is the inhibition of p53-linked transcription [40]. It was found that while serine phosphorylation is required for the negative regulation of FZDs, an alanine mutation inhibits the RNF43 negative control function, inhibiting FZD ubiquitination and degradation, thus leading to enhanced Wnt signaling. In contrast, such modification had no impact on the inhibition of p53-mediated transcription. The serine triplet mutants work together with active Ras to induce a cancer-associated pathway of poor diagnosis in colon cancer [41]. Amazingly, in the presence of phospho-mimetics, RNF43 restored the negative regulation of FZDs, inducing their degradation and preventing Wnt signaling. Additionally, this inhibited the oncogenic Ras-RNF43-mediated cancerous status. Overall, the serine phosphorylation step designates the importance of RNF43 and highlights a multistep mechanism of the cancerous Wnt-RNF43-p53 axis.

## 4. RNF43 Mutants

RNF43 effectively inhibits Wnt signals in healthy circumstances, and numerous genetic mutations in *RNF43* present in many cancers delineate a tumor suppressor type of carcinogenesis. Cancer-related RNF43 mutations promote the activation of β-catenin signaling through the aberrant rise in Wnt receptors at the cell surface membrane. Inactivating endogenous mutations in RNF43 have been found in tumors of the gastrointestinal (GI) system and endometrial and ovarian cancer [23,26]. In fact, the detection of *RNF43* mutations in the blood of cancer patients provides a wealth of information that can be used for the selection of patients that might benefit the most from anti-Wnt therapy [42]. This is because patients comprising *RNF43* mutations are highly dependent on Wnt/β-catenin signaling due to impaired FZD-LRP6 homeostasis. This enhances sensitivity to the Wnt pathway and allows for the identification of a patient population that could gain from upstream Wnt/β-catenin therapies while avoiding the downstream Wnt/β-catenin signaling pathway, which is characterized by abundant mutations [43]. Among current Wnt/β-catenin therapies are porcupine (PORCN) inhibitors and anti-FZD-blocking antibodies, such as OMP-18R5. The most common mutation affecting downstream components in the aberrant cancer Wnt/β-catenin signaling pathway is seen in the gene-encoding adenomatous polyposis coli (APC), with such mutations being seen in RNF43-, ZNRF3-, and RSPO3-mediated cancers [23,26,44,45]. Indeed, an ongoing challenge in precision medicine is how to allocate driver mutations in central cancer genes. The over-expression of RSPO3 in colon cancer is due to *RSPO3* fusion as a result of DNA translocation [44]. While *RSPO3* fusion can be detected through PCR analysis, mutations in *RNF43* are more intricate and require NextGen DNA sequencing for the detection of ample and variable mutations that appear throughout the entire RNF43 sequence. Among these are missense, truncation, and insertion–deletion (indel) mutations [46,47]. In fact, it is important to determine which are “driver” mutations in carcinogenesis, as opposed to “bystander” mutations that do not contribute to the malignant process.

Overall, the RNF43 mutational landscape was prepared by site-directed mutagenesis [45] inserted to the RING or phospho—switches act to inhibit RNF43 negative regulation, as these are needed for the ubiquitination function [39,45]. Yet, it appears that mutations in the N-terminal extracellular region such as I48T, L82S, and R127P act to inhibit RNF43 function and act as dominant negative (DN) mutations. According to the excellent review by Tsukiyama T [48], these mutants act to interrupt the RNF43 internal cell trafficking, causing a pile in the ER [13]. Under this condition of being piled up, RNF43 CK1 can no longer phosphorylate the mutant RNF43 (no phosphorylation switch ON) and, furthermore, they inhibit the formation of *wt* RNF43. Mutant R127P acts as an oncoprotein and, extraordinarily, the phosphorylation mimetic 3SD when introduced (a signal for turn on) converts it back to a tumor suppressor, even when remains in the ER [39]. RNF43 mutants that are unable to degrade FZDs represent accumulated cell surface FZDs, which are highly dependent on the Wnt ligand and, therefore, are sensitive to PORCN inhibitors. The R117fs mutation is a truncation mutation. This is the second most frequent hot spot mutation that retains the PA binding domain but lacks most other domains, including the transmembrane domain and the cytoplasmic tail. Mutant R519X acts in a Wnt-independent manner. It acts via the stabilization of RNF43 association with CK1, leading to β-catenin stabilization through the disassembly of the “destruction complex”, regardless of Wnt ligands. Therefore, this mutant is not sensitive to PORCN inhibitors. The most frequent hot spot mutation R659fs has no impact on Wnt signaling [30]. Overall, the choice of therapy drug should be made depending on the type of RNF43 mutation expressed [2,45,48] (Table 1).

RNF43 comprises the extracellular regions that contain a protease-associated (PA) domain that associates with RSPOs [49] and FZDs [39], a single-pass transmembrane (TM) domain, and an intracellular RING domain, with the enzymatic E3 ubiquitin ligase domain residing in the C-terminal portion. Significantly, mutations affecting the RING and PA domains of RNF43 were detected at a level higher than would be predicted coincidently [45]. This may point to an intentional selection toward cancer. Overall, two main hot spots were assigned as hallmarks of *RNF43* mutations in colon cancer, namely, the R117 frame shift (fs), affecting the N-terminal region of the protein, and the G659fs truncation mutation, affecting the C-terminal domain. While, in general, truncation and missense mutations indicate *RNF43* LOF, the impact of the most frequently reported mutation, p.G659fs, remains controversial. It was recently reported that such mutation does not have an impact on β-catenin signaling, which remains unaffected [50]. This suggests that the behavior of mutant RNF43 should be carefully analyzed in candidates targeting RNF43 so as to avoid referring to N-terminal RNF43 mutants as solely being the result of driver mutations.

Serrated polyposis syndrome (SPS) is now recognized as the precursor to one-third of all CRCs. Assessment of RNF43 mutation status in seventy-four selected SPS families allocated two rare missense variants, c.443C > G (p.A148G) and c.640C > G (p.L214V), which predicted risk by in silico algorithms in two families [51]. Sanger sequencing in ninety-six unrelated patients with SPS in Spain [52] identified two variants: c.394C > T (p.R132*) and c.1821G > A. Overall, an RNF43 mutation in SPS is rare.

## 5. RNF43 Not Only Acts on FZDs but Also Additional Cancer Driver Partners

**Figure 4:**
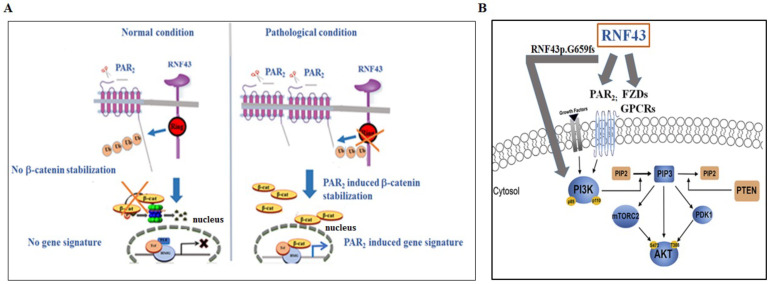
Proposed model for PAR_2_-induced β-catenin stabilization and targets of RNF43. (**A**) PAR_2_ is a target of RNF43, independent of Wnts. A scheme showing the proposed mechanism whereby PAR_2_ markedly induces the β-catenin signaling pathway. In the presence of RNF43, PAR_2_ is degraded, such that no β-catenin stabilization occurs. When RNF43 is ubiquitinated and degraded, abundant levels of membrane PAR_2_ are obtained. Under such conditions, facilitated β-catenin stabilization takes place, and an increased gene signature is observed. (**B**) Targets of RNF43. The E3 ubiquitin ligase RNF43 acts to degrade FZDs and PAR_2_. An additional partner affected by RNF43G659fs is the PI3K/mTOR pathway.

Despite the growing acknowledgment of the contribution of GPCRs to a wide array of physiological and disease functions, there are currently limited GPCR-based cancer drugs available. In addition, the understanding of the post-translational regulation of GPCRs is incomplete. Importantly, GPCRs are centrally involved in many aspects of tumorigenesis, including proliferation, invasion, survival at secondary sites, and several cancer-associated signaling pathways. As a sub-family of GPCRs, FZD receptors play a pivotal role in cancer. Other GPCRs that are involved in epithelial malignancies were also found to induce the stabilization of β-catenin. These include lysophosphatidic acid receptors (LPAs) [53,54], endothelin receptor A (ETR A) activated by ET-1 [55], a prostaglandin receptor (EP2) activated by COX2 [56], and PTHR [57]. Protease-activated receptors (PARs) form a family of four members (PAR1-4) that are potent inducers of β-catenin stabilization. We, as well as others, have demonstrated that PAR_1_ plays a pivotal role in breast cancer invasion and placenta anchorage to the uterus decidua during the first trimester of pregnancy [58,59]. Our original finding linking PARs to β-catenin accumulation was primarily conducted in the mammary glands. In a transgenic mouse model over-expressing *PAR1* targeted to the mammary glands, we showed that the tissues exhibited advanced hyperplasia, characterized by a dense network of ductal side branching and accelerated proliferation. We noticed a striking stabilization of β-catenin localized to the cell nucleus in *PAR1* transgenic mice compared with mammary glands in the age-matched *wt* mice [60,61]. This finding paved the way to explore β-catenin accumulation initiated via PAR family members. Similarly, we found that PAR_2_ and PAR_4_ individually potently induced β-catenin stabilization and transcriptional activity [62,63]. Overall, PAR_1_, PAR_2_, and PAR_4_ oncogenes are powerful inducers of nuclear β-catenin and act via the recruitment of LRP5/6 co-receptors in both the malignant and physiological invasion process of placenta anchorage to the uterus decidua [64,65] (Figure 4A).

PAR_2_-induced β-catenin stabilization was observed in the presence of Wnt/FZD inhibitors [66], namely, either LGK 974, a Wnt porcupine inhibitor, or secreted frizzled-related protein (sFRP5), an FZD inhibitor. Therefore, we considered the consequences of the PAR_2_-induced negative regulation of β-catenin stabilization, independent of Wnts. We demonstrated that biotin-labeled cell surface PAR_2_ was strongly ubiquitinated and degraded in the presence of RNF43, as shown by assessing the levels of cell surface biotin-labeled PAR_2_ detected using Western blots and FACS analyses [66]. Consequently, powerful inhibition of β-catenin stabilization and TOP*flash* transcriptional activity were obtained. In this manner, a new target for RNF43-mediated degradation, namely, PAR_2_/*f2rl1* GPCRs, was described (as shown in Figure 1). Importantly, PAR_2_ is a potent oncogene that plays a central role in diverse epithelial malignancies, including colon [66,67], breast [68,69,70], and ovarian cancers [71,72]. The E3 ubiquitin ligase RNF43 acts on PAR_2_, similar to its homolog ZNRF3, which negatively regulates the turnover of the Wnt receptors, the FZD and LRP6. Notably, PAR_2_ acts with LRP6 as a co-receptor to stabilize β-catenin [64]. Whether the association between RNF43 and PAR_2_ is mediated via DVL as an adaptor for the FZD [10] remains to be determined. As with FZDs, the turnover of PAR_2_ is rescued by the R-spondin–LGR5 axis. The modulation of *RNF43* gene levels showed that by silencing *rnf43* in aggressive cancer cells, sustained surface expression of endogenous PAR_2_ was observed [66].

The intestine tissue is characterized histologically and comprises villi and crypts. Intestinal stem cells (ISCs) are generated in the crypts and following their maturation, they move to the villi. ISCs together with Paneth cells form a niche whereby Paneth cells support stem cell activity by secreting Wnt3a [38]. RNF43 is expressed in the crypt base and resides there. RNAscope in situ hybridization analysis of PAR_2_ localization in the intestine tissues showed a widespread expression throughout the crypts and villi [66]. PAR_2_ most likely interacts with RNF43 within the crypt, whereby RNF43 is localized. The role of PAR_2_ in the villi remains to be studied.

RNF43 p.G659fs, the most frequently (Figure 3B) reported mutant in several cancers, is capable of enhancing cell growth independent of Wnt signaling [73,74]. A library of drug screening assays revealed that cells expressing RNF43p.G659 mutations can be attenuated by the inhibition of PI3K signaling [75]. While the role of RNF43p.G659fs is debatable [45,50], the rate of RNF43p.G659fs appearance is significantly higher than that of the native protein. Indeed, the level of expression seen is greater than would be envisioned by a simple coincident occurrence in colon cancer, pointing to a positive and meaningful cancer selection [26]. Fang and co-workers demonstrated that RNF43p.G659fs exhibits oncogenic activity. The screening of a therapy drug library showed that PI3K/mTOR inhibitors were effective at attenuating tumor models exhibiting RNF43p.G659fs. These showed effectiveness compared with PORCN inhibitors, which had no effect on CRC organoids carrying the RNF43p.G659fs mutation [75]. In summary, PAR_2_*/f2rl1* represents an additional target for RNF43, and the PI3K/mTOR pathway offers a target for RNF43 p.G659fs.

## 6. PAR_2_ Is a Favorable Target for Cancer Therapy

In spite of the continuous progress in cancer treatment, cancer patients often develop resistance to chemotherapy, which considerably influences their survival [76]. To meet the challenges ahead, there is a continuous and pressing need to find new and favorable therapeutic drug targets. Most pharmaceuticals currently in use target GPCRs, based on their involvement in many physiological and pathological processes. Indeed, GPCRs are becoming more acknowledged as valuable targets for cancer therapy [77,78]. The TCGA and GTEx databases show that PAR_2_*/f2rl1* is significantly over-expressed in many types of epithelial malignancies, such as colon [79], breast [80], and ovarian cancers [71].

Several PAR_2_ antagonists have been developed. Small molecules by AstraZeneca (e.g., AZ8848 and AZ3451 [81,82]), as well as I-191, I-287, and I-343 generated by Vertex [82,83,84], show promising outcomes for pain and peripheral inflammation in pre-clinical models. Another strategy being taken involves developing small molecules that mimic the internal activating ligand sequence immediately after the PAR_2_ N-terminal cleavage site. This led to the development of the high-affinity peptidomimetic 2-furoyl-LIGRLO-NH_2_[2fLIGRLO-NH_2_] [85]. Modification of these peptide agonists gave rise to several peptidomimetic inhibitors, such as GB88 [86] and K-14585, both of which inhibit G-protein signaling and were shown to affect inflammation [87,88]. Additional modification of 2f-LIGRLO-NH_2_ produced compound 391 (C391) [89], which uniquely differs from similar antagonists, such as GB88, AZ8838, and AZ3451, in that C391 only needs a minimal pre-incubation period to affect Ca^2+^- and PAR_2_-dependent signaling (e.g., MAPK) pathways, potently inhibiting the activation-dependent function of PAR_2_ in vitro and in vivo.

Pepducins are cell-penetrating peptides directed to the intracellular loops of a targeted receptor and are composed of a lipidated moiety. Once they penetrate the membrane, they bind to the receptor intracellular loop, where the GPCR associates with a G-protein. Pepducins are considered a class of allosteric antagonists. PZ-235, also named P2pal-18S and initially generated by the Kuliopulos group, was directed to the third loop of PAR_2_ and was tested for PAR_2_-induced inflammation [90]. This compound suppressed mast cell tryptase and trypsin-induced signaling in neutrophils and colon cancer cells. It also potently inhibited skin inflammation and itch in both acute and chronic models of dermatitis [91].

We have introduced another PAR-based therapy, namely, P*c*(4-4), directed toward the PAR pleckstrin homology (PH)-binding motifs residing within PAR C-terminal tails [62,92]. P*c*(4-4) effectively inhibits PAR_2_ and PAR_4_ oncogenic activities [65,71]. The PH domain within signal proteins is mainly recognized via its structural characteristics based on a seven-stranded β-sandwich and a C-terminal α-helix. PH domains in signal proteins are not identical in terms of their primary sequences. Rather, their similarity comes from the superfold assembly, which principally represents a stable structural scaffold. As such, upon activation, PARs recruit Akt/PKB signal protein via the PH domain or recruit Etk/Bmx or Vav3, as Gab1 or Sos1. P*c*(4-4) effectively inhibits PAR-Akt-PH association, cell migration, and invasion in vitro and causes inhibition of tumor growth in vivo in mouse models of ovarian [76] and colon cancers [65].

Every year, scores of new cancer patients are diagnosed. In the USA alone, 297,790 new invasive breast cancer patients were diagnosed in 2023. For colon cancer, 153,000 new individuals were diagnosed in the USA, out of which 52,550 will die. In total, nearly 500,000 individuals in the USA in 2023 alone were diagnosed with these two types of cancer. The P*c*(4-4) compound is uniquely effective not only toward PAR-induced tumors; it also potently inhibits EGFR, which is the main target of cancer therapy drugs. Certainly, among patients that can benefit from P*c*(4-4) are those with tumors expressing EGFR that develop resistance to current therapies. Likewise, individuals with breast cancer diagnosed at a triple-negative stage with *Her2/Neu* that develop resistance to current treatments could gain from P*c*(4-4) treatment.

## 7. Concluding Remarks

Dysregulation of the Wnt signaling pathway has been associated with cancer, among other diseases Hence, multiple layers of control mechanisms that tightly regulate Wnt signaling and suppress Wnt activity are significant. The negative layer of regulation by RNF43/ZNRF3 considerably impacts the Wnt pathway and the development of therapy plans [92]. The selection of a patient population that will best respond to a targeted therapy is an ongoing challenge in cancer clinics. Mutations in *RNF43*, identified in blood samples from colon, ovary, pancreas, stomach, and endometrial cancer patients, provide an informative guideline for the selection of a patient population that will positively respond to a given drug. RNF43 mutants with impaired E3 ligase function exhibit high dependency on the Wnt/FZD, PAR_2_, and the PI3K/mTOR signaling pathway, all of which are onco-targets affected by RNF43, as shown in Figure 4B. Therefore, the development of therapeutic drugs that effectively block these essential cancer targets is both timely and necessary.

## Figures and Tables

**Table 1:** Key mutations in RNF43.

RNF43 Mutation	β-Catenin Accumulation	Wnt Ligand Dependent	PORCN Inhibitors
R117 fs	−	−	−
659 fs	+	−	−
I48T	+	+	+
L82S	+	+	+
R127P	+	+	+
H292/295R	+	+	+
S474P/475Y/478P	+	+	+
R519X	+	−	−

Key mutations in RNF43 are shown. Consequently, β-catenin accumulation status and Wnt ligand dependency are indicated. As a result, the envisioned response to PORCN inhibitors is shown.
